# Glioneuronal tumor in an autosomal-dominant polycystic kidney disease patient: a case report and literature review

**DOI:** 10.1097/MS9.0000000000002265

**Published:** 2024-06-10

**Authors:** Salem K. Qupp, Mohammad M. Zeidan, Hafez Nimer

**Affiliations:** a Faculty of Medicine, Al-Quds University, Jerusalem, Palestine; b Department of Neurosurgery, HClinic, Ramallah, Palestine, Palestine

**Keywords:** ADPKD, case report, glioma, glioneuronal tumor, polycystic kidney disease

## Abstract

**Introduction:**

The association between primary brain tumors, such as glioneuronal tumors, with autosomal-dominant polycystic kidney disease (ADPKD) remains poorly understood, with only two cases reported excluding this one. This case of an ADPKD patient diagnosed with a rosette-forming glioneuronal tumor highlights an exceptionally rare potential association warranting further investigation.

**Case presentation:**

A 28-year-old male with ADPKD presented with progressive ataxia, dizziness, and headache. MRI revealed a cerebellar mass and obstructive hydrocephalus. Surgical resection and histopathological examination confirmed the diagnosis of a rosette-forming glioneuronal tumor. Postoperatively, the patient showed significant symptom improvement.

**Discussion:**

The interplay between genetics and glioneuronal development is complex and underexplored. While most glioneuronal arise sporadically, rare genetic syndromes may predispose individuals to these tumors. Additionally, although more than 70 cases of ADPKD with concurrent tumors were reported, the literature on this specific association remains limited.

**Conclusion:**

This case underscores the need for heightened awareness of potential associations between ADPKD and tumors such as glioneuronal tumors. With limited literature on this subject, further research is imperative to understand the underlying mechanisms and clinical implications. Enhancing our knowledge in this area can improve patient outcomes and management strategies.

## Introduction

Highlights

- Autosomal-dominant polycystic kidney disease (ADPKD) patients may have an increased risk of various Tumors, suggesting a link between the disease and Tumors’ development.
- Primary brain tumors, such as glioneuronal tumors, are exceptionally rare in ADPKD, with few cases documented.
- Further research is needed to explore the potential association between ADPKD and glioneuronal tumors and its clinical implications.

Glioneuronal tumors are rare primary brain tumors that comprise neural and glial elements in heterogeneous proportions^1,2^. More than 50% of patients exhibit symptoms such as headache^3^, hydrocephalus^4^, and focal neurological deficits^5^. Subtypes encompass central, extraventricular, and lipo-neurocytoma, desmoplastic infantile astrocytoma, ganglioglioma, and rosette-forming glioneuronal tumor (RGNT). Understanding their pathology and molecular profiles is crucial for their clinical significance and guiding research^1,2^.

With less than 50 reported cases^6^, RGNT is a rare, slow-growing tumor, predominantly affecting the fourth ventricular region, primarily in young adults. It is characterized by a biphasic histology with neurocytic rosettes and glial components. RGNTs typically present with symptoms such as headache and ataxia and are treated surgically^7^.

Autosomal-dominant polycystic kidney disease (ADPKD) is a hereditary disorder characterized by cyst formation in the kidneys and other organs, affecting ~1 in 400–1 in 1000 live births^8^. Cyst formation starts early in life, with macroscopic cysts potentially becoming observable during childhood^9^. It mostly results from mutations in the polycystic kidney disease 1 (PKD1) or polycystic kidney disease 2 (PKD2) genes^10^. Approximately 9% of cases arise from de novo, pathogenic genetic variations^11^. ADPKD can lead to an irreversible decline in kidney function, accompanied by complications such as hypertension, hematuria, proteinuria, and renal insufficiency. This disease is also associated with extrarenal complications, including cerebral aneurysms, hepatic and pancreatic cysts, and cardiac valve disease^10^. To date, data elucidating the relationship between overall cancer risk and polycystic kidney disease remains scarce^12^.

Herein, we present the first documented case of an ADPKD patient diagnosed with a posterior fossa RGNT, marking the third reported primary brain tumor in an ADPKD patient. This case report aims to highlight this rare association, suggesting that there may be an underlying link between ADPKD and primary brain tumors including glioneuronal tumors that warrants further investigation. Additionally, we conducted a review of cases involving concurrent tumors in patients diagnosed with ADPKD. This could have important implications for understanding the disease mechanisms and improving the management of patients with ADPKD.

### Case report

A 28-year-old male patient was admitted to our hospital with a 1-week history of progressively worsening ataxia, dizziness, and headache. The patient had no history of trauma or recent illness but had a significant medical and familial history of ADPKD.

Upon ICU admission, the patient was conscious, alert (Glasgow Coma Scale 15/15), afebrile, and hemodynamically stable. Neurological examination revealed intact cranial nerves and voluntary movement, but signs of ataxia and mild lower limb weakness were present.

**Figure 1:**
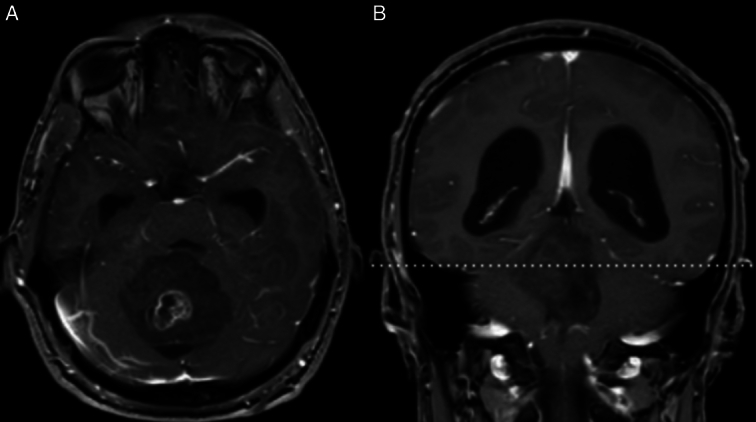
(A) Horizontal T1-weighted MRI scan of the head revealing the presence of a cerebellar mass; (B) coronal T1-weighted MRI scan of the head, highlighting both obstructive hydrocephalus and the cerebellar mass.

Brain computed tomography revealed a large mass (~5 cm) in the midline position of the posterior fossa, associated with moderate to severe obstructive hydrocephalus due to obstructing cerebrospinal fluid flow. Subsequent brain MRI confirmed a diagnosis of a posterior fossa tumor with additional findings of obstructive hydrocephalus. (Fig. 1).

Following the neurosurgical evaluation, the patient underwent midline suboccipital craniotomy with microscopic maximal safe resection of the midline cerebellar tumor to achieve complete excision while minimizing the risk of neurological deficits. Additionally, an external ventricular drain was inserted to manage obstructive hydrocephalus. Intraoperative neurophysiological monitoring was used to assess the integrity of the neural pathways and guide surgical decision-making. The procedure successfully achieved gross total mass resection with no intraoperative complications.

Histopathological examination of the resected tumor tissue revealed a low-grade, well-differentiated rosette-forming glioneuronal neoplasm.

The patient was closely monitored in the intensive care unit following the surgery. He tolerated the procedure well and demonstrated significant improvement in symptoms. An external ventricular drain remained in place for postoperative cerebrospinal fluid drainage and intracranial pressure monitoring.

Serial neurological assessments revealed progressive improvement in lower limb weakness and gait stability.

## Discussion

The relationship between genetics and glioneuronal tumor development is complex, involving both inherited syndromes and somatic mutations. While most glioneuronal tumors arise sporadically, certain rare genetic syndromes may predispose individuals to these tumors due to specific mutations. RGNTs have been reported to be linked to neurofibromatosis type 1 (NF1) gene mutations^13^. RGNT has also been documented in an individual with dysgenetic trichorhinopharyngeal Type 1 syndrome^14^. Azam *et al*.^15^ reported a case of glioneuronal tumor in a patient with Lynch syndrome with features of somatic and germline MSH2 mutations. Moreover, tuberous sclerosis has been linked to many brain lesions including glioneuronal tumors^16^. The association between ADPKD and primary brain tumors including glioneuronal tumors, however, is not described in the literature. Understanding these genetic associations informs personalized treatment strategies tailored to individual tumor profiles, offering opportunities for improved outcomes and early intervention in high-risk individuals.

ADPKD, marked by cyst formation in both kidneys, leads to a decline in renal function, hematuria, and pain, frequently exhibits various extrarenal symptoms. These include cysts in diverse organs, such as the liver, seminal vesicle, pancreas, arachnoid membrane, and spinal meninges, along with connective tissue abnormalities, such as mitral valve prolapse, intracranial aneurysms, diverticular disease, and abdominal hernia. Cardiovascular issues such as hypertension and left ventricular hypertrophy are also prevalent^17^.

ADPKD arises from mutations in several genes, with three main mutations being identified. PKD1, found on chromosome 16p13.3, is responsible for 85% of cases, whereas PKD2, located on chromosome 4q21, contributes to 15% of cases. GANAB mutations are implicated in approximately 1% of patients with ADPKD, often with variable polycystic liver involvement. PKD1 and PKD2 mutations exhibit similar phenotypes, but PKD1 typically leads to earlier renal replacement therapy dependency, affecting approximately 50% of patients by the age of 60 years. PKD2 mutations tend to manifest in older individuals, presenting with milder symptoms, fewer kidney cysts, delayed hypertension onset, and less frequent end-stage renal disease than PKD1 mutations^18,19^.

As of now, the available evidence regarding the pathological interaction between PKD and tumors remains uncertain and subject to debate. However, more than 70 cases have been reported of ADPKD patients with concurrent tumors providing insights into this potential interaction. A population-based study revealed that the association between PKD and cancer, particularly renal cell carcinoma, is stronger in individuals with PKD than in those without PKD. Moreover, patients with PKD also exhibit an increased risk of liver, pancreatic, and colon cancers. This finding suggests a potential link between the mTOR pathway and carcinogenesis in PKD, as mTOR inhibitors have been utilized to manage cyst formation in patients with PKD^12^.

A patient with ADPKD developed papillary renal cell carcinoma (pRCC). Genetic analysis revealed a somatic mutation in the MET proto-oncogene specific to the pRCC, distinct from ADPKD-associated mutations. Increased MET expression in the pRCC suggests a unique pathway in cancer development. While a constitutional PKD1 germline mutation was present in the tumor, somatic mutations in PKD1 were exclusive to renal cysts^20^.

A review of 53 cases of ADPKD adult patients that reported concurrent tumors revealed multiple types of tumors, including renal cell carcinoma, pancreatic cystadenocarcinoma, intrahepatic cholangiocarcinoma, primary carcinoid tumor, transitional cell carcinoma, urothelial carcinoma, pancreatic adenocarcinoma, central nervous system lymphoma, multiple myeloma, renal papillary adenoma, adrenal incidentaloma, and two cases of glioma, glioblastoma, and astrocytoma^16^. Moreover, a study by Grime *et al*.^21^ reviewed ten cases of concurrent gastric cancer in ADPKD patients.

Friend and colleagues described four patients with a history of ADPKD who were diagnosed with malignancies during childhood, without any additional known mutations suggesting a genetic predisposition to cancer development. Among these patients, two were siblings diagnosed with hereditary testicular germ cell tumors, while the remaining two were diagnosed with rhabdoid tumors of the kidney and perivascular epithelioid cell tumors with liver metastases^22^. Cases of Wilms tumor in PKD were also reported. Thankamony *et al*.^23^ reported a child with bilateral PKD and PHACE syndrome who developed Wilms tumor in the right kidney at the age of 17 months. Gucev *et al*.^24^ reported an 8.5-year-old patient with WAGR syndrome, which includes Wilms tumor, and bilateral PKD. Zina *et al*.^25^ also reported an ADPKD patient who was accidentally diagnosed with Wilms tumor at two years of age during an abdominal MRI.

Excluding this case, only two cases reported primary brain tumor in ADPKD patients. In the first case, a 57-year-old man presented with a cystic glioblastoma in the right temporal lobe, alongside multiple kidney and liver cysts characteristic of ADPKD, the patient underwent gross total resection followed by radiotherapy and chemotherapy. In the second case, a 27-year-old man with a non-enhancing glioma in the right parietal lobe, also accompanied by kidney and liver cysts indicative of ADPKD, the patient underwent subtotal resection followed by radiotherapy^16^. In our case, the 28-year-old patient presented with kidney cysts without liver involvement and a midline posterior fossa RGNT, gross total resection of the tumor was done. These cases highlight the rare coexistence of ADPKD and primary brain tumors, suggesting a potential genetic or molecular link between the two conditions.

This case report is constrained by the limited availability of comprehensive genetic profiling facilities within the hospital, which impeded the thorough exploration of potential genetic associations. Additionally, the scarcity of documented cases linking primary brain tumors with glial features to ADPKD in medical literature may undermine the perceived significance of the association. However, dismissing these findings as mere coincidences may overlook the potentially profound impact of such an association, particularly in the dominant form, on neurological prognosis and patient outcomes. Consequently, further studies are needed to thoroughly investigate the relationship between ADPKD and glial and glioneuronal tumors.

## Conclusion

Multiple cases have reported concurrent tumors in ADPKD patients, which underscores the importance of considering additional factors beyond the classic manifestations of ADPKD. This case report documents the rare occurrence of a glioneuronal tumor in a patient with ADPKD, contributing to the very limited literature on primary brain tumors in ADPKD patients. Our findings underscore the potential for an association between ADPKD and primary brain tumors, highlighting the importance of recognizing such presentations in clinical practice. Further studies are needed to elucidate the underlying mechanisms and clinical implications of this potential association.

## Ethical approval

This study is exempt from ethical approval in our institution.

## Consent

Written informed consent for the data and pictures was obtained from the patient and is available upon request from the Editors-in-Chief.

## Source of funding

The authors declare that the writing and publishing of this manuscript were not funded by any organization

## Author contribution

Writing of the manuscript: S.K.Q. and M.Z. Literature review: S.Q. Investigation and imaging description: H.N. Reviewed and edited the manuscript: S.K.Q., M.Z., and H.N.

## Conflicts of interest disclosure

The authors declare no conflicts of interest.

## Research registration unique identifying number (UIN)

This manuscript is a case report and not a human study; therefore does not need to be registered.

## Guarantor

Hafez Nimer.

## Data availability statement

This submission is a case report and therefore does not include any results derived from research data.

## Provenance and peer review

This paper was not invited.
